# Influence of sex on sympathetic vasomotor outflow responses to passive leg raising in young individuals

**DOI:** 10.1186/s12576-024-00909-4

**Published:** 2024-03-18

**Authors:** Keisho Katayama, Kana Shiozawa, Jordan B. Lee, Natsuki Seo, Haruna Kondo, Mitsuru Saito, Koji Ishida, Philip J. Millar, Ryoichi Banno, Shigehiko Ogoh

**Affiliations:** 1 Research Center of Health, Physical Fitness and Sports, Nagoya University, Nagoya, 464-8601 Japan; 2 Graduate School of Medicine, Nagoya University, Nagoya, 464-8601 Japan; 3 Japan Society for the Promotion of Science, Tokyo, Japan; 4 Department of Physiology and Pharmacology, University of Calgary, Calgary, Canada; 5 Applied Physiology Laboratory, Toyota Technological Institute, Nagoya, Japan; 6 Department of Human Health and Nutritional Sciences, University of Guelph, Guelph, Canada; 7 Department of Biomedical Engineering, Toyo University, Kawagoe, Japan

**Keywords:** Cardiopulmonary baroreflex, Sex differences, MSNA, Sympathetic nerve activity

## Abstract

The purpose of this study was to clarify sex differences in the inhibition of sympathetic vasomotor outflow which is caused by the loading of cardiopulmonary baroreceptors. Ten young males and ten age-matched females participated. The participants underwent a passive leg raising (PLR) test wherein they were positioned supine (baseline, 0º), and their lower limbs were lifted passively at 10º, 20º, 30º, and 40º. Each angle lasted for 3 min. Muscle sympathetic nerve activity (MSNA) was recorded via microneurography of the left radial nerve. Baseline MSNA was lower in females compared to males. MSNA burst frequency was decreased during the PLR in both males (− 6.2 ± 0.4 bursts/min at 40º) and females (− 6.5 ± 0.4 bursts/min at 40º), but no significant difference was detected between the two groups (*P* = 0.61). These results suggest that sex has minimal influence on the inhibition of sympathetic vasomotor outflow during the loading of cardiopulmonary baroreceptors in young individuals.

## Background

The cardiopulmonary baroreflex plays a crucial role in maintaining hemodynamic homeostasis, in large part through precise regulation of sympathetic vasomotor outflow [6, 31, 44]. The majority of research on the cardiopulmonary baroreflex has focused on neural adjustments during orthostatic stress. Indeed, sympathetic vasomotor outflow directed to the skeletal muscle vasculature (muscle sympathetic nerve activity: MSNA) increases during low levels of lower body negative pressure (LBNP) [42, 48] or mild experimental hemorrhage [40], which induces decreases in central blood volume (CBV) and central venous pressure (CVP), thereby unloading the cardiopulmonary baroreceptors [31]. In contrast, though less extensively studied, increases in CBV and CVP, which load the cardiopulmonary baroreceptors, evoke sympathoinhibition. Instances of such responses include decreased MSNA and increased forearm blood flow observed during passive elevation of the legs [16, 30, 41], low levels of head-down tilt [44], or mild lower body positive pressure [13, 25]. Loading of the cardiopulmonary baroreceptors also contributes significantly to sympathoinhibition during low-intensity dynamic exercise [8, 26] by enhancing venous return and, consequently increasing CBV and CVP via muscle pump.

Physical and physiological differences exist between males and females, including different circulating levels of the sex hormones like testosterone and estrogen. These sex-specific differences could potentially impact baroreflex function [11, 19]. Regarding cardiopulmonary baroreceptor unloading, Yang et al. [51] compared changes in MSNA during LBNP between young males and females, and found that increases in MSNA during low levels of LBNP were similar between sexes [51]. This result indicates minimal sex differences in the sympathoexcitatory response during cardiopulmonary baroreceptor unloading. To the best of our knowledge, whether sex differences in the sympathoinhibitory effect of cardiopulmonary baroreceptor loading exist has not been evaluated in humans. Investigating the sex differences in the inhibition of sympathetic vasomotor outflow through the cardiopulmonary baroreflex may generate foundational data on sex-specific influence on sympathetic vasomotor and arterial blood pressure (ABP) regulations during low-intensity dynamic exercise.

We tested the hypothesis that the magnitude of the sympathoinhibitory response to cardiopulmonary baroreceptor loading would not differ by sex. To test this hypothesis, MSNA was measured during passive leg raises (PLR), a method known to load the cardiopulmonary baroreceptors, in young males and age-matched females.

## Methods

### Participants

Fifteen young males and 14 young females were recruited. All participants were free of known diseases and were non-smokers. Females did not take medications, oral contraceptive pills, or other forms of hormonal contraception. Testing for female participants was not standardized to a specific time point within the menstrual cycle. We were unable to obtain or maintain sufficient quality MSNA recordings in nine participants (five males and four females). Consequently, we report data from ten males (age: 22 ± 1 years) and ten females (age: 21 ± 1 years).

### Experimental procedure

All experiments were performed in a temperature-controlled laboratory (22–24 ˚C). On day 1, subjects were familiarized with the measurement apparatus and the PLR test. The participants were placed in a supine position (0º) on a customized bed allowing for movement about the hip joint, and then the lower limbs of the participants were lifted passively in a straight manner to 40º. On day 2, participants performed the PLR test with MSNA measurement (MSNA trial). To prevent arm movement artifacts, the arms were fixed using a vacuum splint (E-13; Okada Medical Supply, Tokyo, Japan). First, the participants rested in a supine position (0º) for 5 min (baseline). Then, the lower limbs were lifted passively and gradually at 10º, 20º, 30º, and 40º and were returned to 0º. Each angle lasted for 3 min. On day 3, participants completed the same PLR test as day 2, but peripheral venous pressure was measured as estimated central venous pressure (eCVP; eCVP trial). MSNA and eCVP trials were conducted with 4- to 7-day interval between them.

### Experimental measurements and instrumentation

#### Cardiovascular variables

An electrocardiogram (ECG) was recorded using a three-lead electrocardiograph (AB-621; Nihon Kohden, Tokyo, Japan). ABP was measured on a beat-to-beat basis using servo-controlled finger photoplethysmography (Finometer; Finapres Medical Systems BV, Amsterdam, The Netherlands) on the middle finger of the right hand. To validate absolute arterial blood pressure values from the Finometer, an automated sphygmomanometer (HEM-907, Omron, Kyoto, Japan) recorded ABP on the brachial artery of the right arm before baseline measurements.

#### Muscle sympathetic nerve activity

Multiunit MSNA was recorded by the standard microneurographic technique, similar to that in our previous studies [21, 24, 27, 28]. A unipolar tungsten microelectrode was placed into the left radial nerve at the posterior aspect of the middle humerus [20, 25, 27], guided by ultrasound imaging [5]. Neural signals were amplified (DAM50; World Precision Instruments, Sarasota, FL, USA), filtered (bandwidth 700–2,000 Hz), rectified, and integrated (time constant 0.1 s) (299; Intercross, Tokyo, Japan) to obtain mean voltage neurograms. MSNA recordings were identified by their pulse synchronous burst pattern and increased burst frequency to an end-expiratory breath hold without any responses to arousal or skin stroking [7, 9, 47].

#### Estimated central venous pressure

The central venous pressure was estimated from the peripheral venous pressure (eCVP), which was monitored using a cannula in the right large antecubital vein as in previous studies [6, 15, 27, 33]. The venous catheter was connected to a pressure transducer (AP-601G, Nihon Kohden, Tokyo, Japan) through a fluid-filled system (PX600F; Edwards Lifesciences Co., Tokyo, Japan). The transducer was calibrated with a manometer before connection to the catheter. The right arm was positioned at the level equal to one-half the transverse chest diameter determined at the fourth rib [45]. All eCVP responses during the PLR test are reported as the changes from baseline values (ΔeCVP) [27, 46].

#### Data acquisition and analysis

ECG, ABP, MSNA, and eCVP were sampled at a frequency of 1000 Hz through an analog-to-digital converter (PowerLab; ADInstruments, Bella Vista, NSW, Australia) and saved to a computer (CF-F8, Panasonic, Osaka, Japan) for off-line analysis. Heart rate (HR) was calculated on R–R intervals recorded from the ECG. Systolic and diastolic ABP (SAP and DAP) were determined from the ABP waveform signal, and mean ABP (MAP) was calculated using the following equation: MAP = (SAP-DAP)/3 + DAP. The MSNA bursts were identified from the mean voltage neurogram using a customized computer program-assisted inspection [21–23, 27, 28], which accounted for the latency from the ECG-R wave to the sympathetic burst [9] and incorporated a signal-to-noise ratio of at least 3:1. For burst amplitude, the bursts during baseline were assigned a mean value of arbitrary units (a.u.) and all MSNA burst amplitudes were expressed as a percentage of this value. MSNA burst frequency (BF; in bursts/min), burst incidence (BI; in bursts/100 heartbeats), and total activity (TA; i.e., mean burst amplitude × burst frequency, in a.u./min) were calculated [20, 25]. All baseline data were averaged over 3-min periods. Variables during the PLR were averaged over 1 min. For statistical analysis, we used the mean values of the last 2 min during each angle. Owing to the baseline differences in MSNA BF, BI, and TA between males and females, the absolute changes from baseline (Δ) were calculated and compared between the two groups.

#### Statistical analysis

Values are expressed as mean ± SE. For all data, the assumption of a normal distribution was verified using the Shapiro–Wilk test. Comparisons of parameters between males and females were performed using Student’s unpaired t-test for variables that were normally distributed, while the Mann–Whitney U-test was used when the distribution was non-normal. Changes in the variables during the PLR test between males and females were compared by two-way ANOVA RM (Group × Angle). Statistical significance was set at *P* < 0.05. Statistical comparisons were performed with SPSS (v.22.0; IBM Japan, Tokyo, Japan) and StatView software (5.0; SAS Institute, Tokyo, Japan).

## Results

### Physical characteristics

Males were taller and heavier than females (males, height: 177 ± 2 cm, body mass: 69 ± 3 kg, females, height 157 ± 2 cm, body mass: 50 ± 3 kg, both *P* < 0.01).

### PLR test

#### Baseline descriptive data

**Fig. 1:**
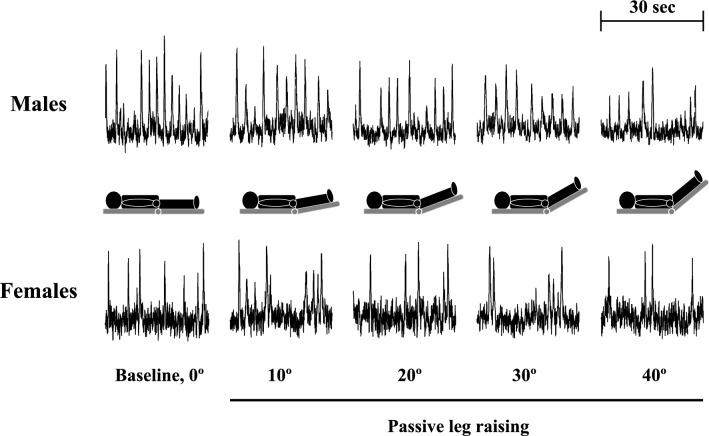
Representative recordings of MSNA at baseline (0º) and during passive leg raising (10º–40º)

**Fig. 2:**
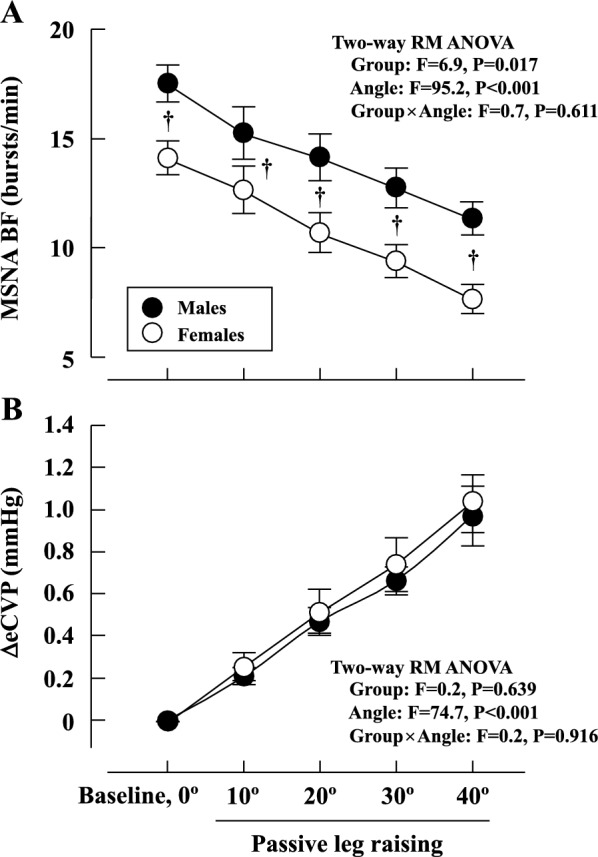
Changes in MSNA BF (**A**) and ΔeCVP (**B**) during the PLR test.^†^*P* < 0.05 males vs. females

A representative MSNA recording is shown in Fig. 1, and mean values of cardiovascular and MSNA variables, and eCVP during the PLR test are shown in Tables 1 and 2, and Fig. 2. There were no differences in any of the HR and ABP values at baseline between the MSNA (day 2) and eCVP (day 3) trials in either group. Thus, we exclusively refer to the HR and ABP data from the MSNA trial. SAP, MAP, MSNA BF, MSNA BI, and MSNA TA were lower in females than males (all *P* < 0.05). In contrast, there were no statistical differences in HR and DAP at baseline between males and females.

**Table 1:** Cardiovascular variables during the PLR test

	Groups	PLR test (angle)					Two-way RM ANOVA
		Baseline, 0º	10º	20º	30º	40º	
HR (beats/min)	Males	61.0 ± 1.6	61.0 ± 2.1	60.7 ± 1.8	61.2 ± 1.6	61.0 ± 1.5	Group: *F* = 0.2, *P* = 0.471 Angle: *F* = 1.2, *P* = 0.314 Group × Angle: *F* = 0.9, *P* = 0.471
	Females	60.2 ± 1.5	60.7 ± 1.5	59.5 ± 1.4	60.1 ± 1.5	59.0 ± 1.3	
SAP (mmHg)	Males	123.1 ± 2.2	122.3 ± 2.5	122.9 ± 2.4	123.0 ± 2.1	123.9 ± 2.1	Group: *F* = 4.9, *P* = 0.041 Angle: *F* = 1.1, *P* = 0.364 Group × Angle: *F* = 1.7, *P* = 0.155
	Females	114.4 ± 2.3^†^	117.8 ± 2.2^†^	117.6 ± 2.2^†^	114.4 ± 2.6^†^	116.3 ± 2.3^†^	
DAP (mmHg)	Males	66.9 ± 1.7	67.7 ± 1.9	66.2 ± 2.4	67.0 ± 2.5	68.2 ± 1.8	Group: *F* = 2.3, *P* = 0.149 Angle: *F* = 1.4, *P* = 0.229 Group × Angle: *F* = 1.1, *P* = 0.379
	Females	62.8 ± 1.4	63.6 ± 1.4	64.5 ± 1.3	62.4 ± 1.6	64.5 ± 1.2	
MAP (mmHg)	Males	85.7 ± 1.6	85.9 ± 1.7	85.1 ± 2.1	85.7 ± 2.1	86.8 ± 1.6	Group: *F* = 4.5, *P* = 0.048 Angle: *F* = 1.6, *P* = 0.176 Group × Angle: *F* = 1.1, *P* = 0.388
	Females	80.0 ± 1.4^†^	81.7 ± 1.4^†^	81.7 ± 1.3^†^	79.8 ± 1.8^†^	81.8 ± 1.3^†^	

Values are mean ± SE. HR, heart rate; SAP, systolic arterial blood pressure; DAP, diastolic arterial blood pressure; MAP, mean arterial blood pressure

^†^*P* < 0.05 vs. Males

**Table 2:** MSNA variables during the PLR test

	Groups	PLR test (angle)					Two-way RM ANOVA
		Baseline, 0º	10º	20º	30º	40º	
ΔMSNA BF (bursts/min)	Males	0.0 ± 0.0	− 2.3 ± 0.6	− 3.4 ± 0.5	− 4.8 ± 0.4	− 6.2 ± 0.4	Group: *F* = 0.1, *P* = 0.833 Angle: *F* = 95.2, P < 0.001 Group × Angle: *F* = 0.7, *P* = 0.611
	Females	0.0 ± 0.0	− 1.5 ± 0.6	− 3.4 ± 0.6	− 4.7 ± 0.5	− 6.5 ± 0.4	
MSNA BI (bursts/100 heartbeats)	Males	29.1 ± 1.9	25.5 ± 2.4	23.8 ± 2.2	21.2 ± 1.8	18.9 ± 1.5	Group: *F* = 5.0, *P* = 0.038 Angle: *F* = 92.2, P < 0.001 Group × Angle: *F* = 0.4, *P* = 0.820
	Females	23.7 ± 1.4^†^	21.0 ± 1.7^†^	18.1 ± 1.4^†^	15.8 ± 1.2^†^	13.1 ± 1.2^†^	
ΔMSNA BI (bursts/100 heartbeats)	Males	0.0 ± 0.0	− 3.7 ± 0.9	− 5.4 ± 1.0	− 8.0 ± 0.7	− 10.2 ± 0.8	Group: *F* < 0.1, *P* = 0.940 Angle: *F* = 92.2, P < 0.001 Group × Angle: *F* = 0.4, *P* = 0.820
	Females	0.0 ± 0.0	− 2.7 ± 0.9	− 5.6 ± 1.1	− 7.9 ± 0.8	− 10.6 ± 0.8	
Normalized MSNA burst amplitude (%)	Males	0.0 ± 0.0	− 15.4 ± 6.9	− 25.9 ± 8.0	− 35.0 ± 5.5	− 41.6 ± 6.8	Group: *F* = 0.4, *P* = 0.515 Angle: *F* = 146.3, *P* < 0.001 Group × Angle: *F* = 0.3, *P* = 0.897
	Females	0.0 ± 0.0	− 26.9 ± 6.6	− 29.2 ± 6.1	− 35.8 ± 6.3	− 42.0 ± 5.7	
MSNA TA (a.u./min)	Males	91.2 ± 11.3	66.5 ± 11.5	54.1 ± 10.7	44.6 ± 8.5	35.4 ± 7.0	Group: *F* = 1.4, *P* = 0.259 Angle: *F* = 73.7, *P* < 0.001 Group × Angle: *F* = 0.3, *P* = 0.896
	Females	74.0 ± 8.1^†^	51.0 ± 9.1^†^	40.7 ± 6.8^†^	32.7 ± 5.5^†^	24.1 ± 4.3^†^	
ΔMSNA TA (a.u./min)	Males	0.0 ± 0.0	− 24.7 ± 5.3	− 37.2 ± 5.7	− 46.6 ± 5.6	− 55.8 ± 6.1	Group: *F* = 0.3, *P* = 0.580 Angle: *F* = 73.7, P < 0.001 Group × Angle: *F* = 0.3, *P* = 0.896
	Females	0.0 ± 0.0	− 23.0 ± 4.0^†^	− 33.3 ± 4.8^†^	− 41.3 ± 6.2^†^	− 49.9 ± 6.0^†^	

Values are mean ± SE. MSNA BF, MSNA burst frequency; MSNA BI, MSNA burst incidence; MSNA TA, MSNA total activity

^†^*P* < 0.05 vs. Males

#### Cardiovascular variables

HR, SAP, DAP, and MAP did not change during the PLR in each group (Table 1). SAP and MAP were lower in females than in males throughout the PLR test (Table 1).

#### MSNA variables

MSNA BF, BI, amplitude, and TA decreased progressively from baseline during PLR in both groups, and MSNA BF, BI, and TA were lower in females than in males throughout the PLR test (Figs. 1, 2A, and Table 2). There were no significant differences in the extent of the decrease in MSNA BF, BI, amplitude and TA during the PLR test between males and females (Fig. 2A and Table 2). Similarly, no statistically significant differences in ΔMSNA BF, BI, and TA during the PLR test were observed between males and females (Table 2).

#### Estimated central venous pressure

The ΔeCVP increased in proportion to the angle during the PLR test in both groups (Fig. 2B). There was no difference in the magnitude of the increase in ΔeCVP during the PLR test between males and females (Fig. 2B).

## Discussion

The novel findings of the present study are as follows: (1) MSNA BF, BI, and TA decreased in proportion to the angle of lower limbs during the PLR test in males and females; (2) the magnitude of the decrease in MSNA exhibited no difference between sexes; (3) eCVP increased linearly in both groups as PLR angles progressed, with no notable difference in the magnitude of eCVP increase (i.e., ΔeCVP) between the two groups; and (4) HR and ABP remained unchanged during the PLR test in both male or female participants. These findings indicate that sex has minimal influence on the sympathoinhibitory response during the PLR test in young individuals. The results of this study provide novel information on the effects of biological sex on sympathetic vasomotor outflow regulation through the cardiopulmonary baroreflex.

### Sex difference in the sympathoinhibitory response during the PLR test

Previously, Yang et al. [51] reported no sex-based differences in the increase in MSNA during low levels of LBNP (~ 10 mmHg) in young, healthy individuals. Their results indicate no sex differences in sympathoexcitation induced by the unloading of cardiopulmonary baroreceptors. Based on the results of their study, we hypothesized there would be no sex difference in the sympathoinhibitory response to cardiopulmonary baroreceptor loading. Consequently, during the PLR test, MSNA BF, BI, and TA decreased gradually in proportion to the lower limb angle in both males and females. However, there were no differences in the changes in MSNA BF, BI, and TA between the two groups (Fig. 2A and Table 2). These findings lead us to conclude that sex minimally influences the cardiopulmonary baroreceptor loading-mediated inhibition of sympathetic vasomotor outflow in young individuals. To our knowledge, this is the first study which investigated the impact of sex on the sympathoinhibitory effect of cardiopulmonary baroreceptor loading. Our findings align with a prior animal study which reported similar decreases in renal sympathetic nerve activity in male and female rats through volume expansion using saline to activate cardiopulmonary baroreceptors [17]. In the present study, eCVP increased in proportion to lower limb angles during the PLR test in both males and females, and the ΔeCVP during the PLR test did not differ between the sexes (Fig. 2B). Thus, it is likely that a similar stimulus was applied to the cardiopulmonary baroreceptors during the PLR test in both groups [27], resulting in comparable levels of MSNA for each angle in both sexes.

Our conclusion that cardiopulmonary baroreflex control of sympathetic vasomotor outflow is not affected by sex in young individuals is based on similar absolute reductions of the MSNA BF, BI, and TA values in males and females (Fig. 2A and Table 2). Whether the absolute or percentage change is more appropriate when investigating physiological responses to a stimulus remains under debate [6, 44]. Given the lower baseline MSNA level in females compared to males (Fig. 2A), it is plausible that the percentage change in MSNA during the PLR test might be more pronounced in females. It has been suggested that the absolute change in (rather than the percentage) MSNA exerts the most influence on target organ responses, thus being the appropriate physiologically significant measure [44] rather than the percentage change. MSNA BF, BI, and TA metrics are related to the plasma noradrenaline concentration [32, 36, 37] and renal noradrenaline spillover [49]. Furthermore, most recent studies [2, 14, 29, 34, 38, 50] comparing MSNA responses to acute physiological stress (i.e., cold pressor test and handgrip exercise) among different groups have reported absolute MSNA changes exclusively. Therefore, it is plausible that the absolute, rather than the percentage change, is of most physiologically significance.

### Limitation and technical consideration

We did not control for the menstrual cycle in female participants. Baseline MSNA changes throughout the menstrual cycle, as reproductive hormones have been shown to influence autonomic control [35]. Previous studies have investigated the effect of menstrual cycle on the arterial baroreflex, but the findings are inconsistent. Minson et al. [35] found that sympathetic baroreflex sensitivity during pharmacological blood pressure perturbations was greater in the mid-luteal phase than in the early follicular phase in young females. In contrast, Fu et al. [12] reported that spontaneous arterial baroreflex sensitivity was not different between the early follicular phase and the mid-luteal phase in young females. These inconsistent results may be attributed to the different methods used to evaluate sympathetic baroreflex sensitivity and/or variable surges in estrogen and progesterone at different time points during the menstrual cycle [11]. In response to orthostatic maneuvers, sympathetic baroreflex sensitivity increased in all individuals and the increment was similar between healthy young males and females during different menstrual phases [3, 12]. To our knowledge, there is no available data concerning the effect of the menstrual cycle on changes in MSNA in response to cardiopulmonary baroreceptor loading, and further study is needed to clarify this issue.

We utilized PLR to simulate cardiopulmonary baroreceptors and compared the changes in MSNA between females and males. The mobilized blood volume during the PLR test may vary depending on anthropometric features [1]. Indeed, males were taller and heavier than females, and thus the changes in blood volume from the lower limbs toward the central part of the body may differ between the two groups. However, there was no difference in the magnitude of the increase in eCVP during PLR test between males and females (Fig. 2B). This result is supported by previous study [4] which measured CVP (right atrial catheter) during PLR in young and older individuals: the young group was taller and heavier than older group, but the increase in CVP induced by PLR was comparable in young and older groups. Thus, we consider that stimulation to the cardiopulmonary baroreceptors during the PLR test was similar in females and males in this study, resulting in comparable levels of MSNA.

### Perspective and significance

The cardiopulmonary baroreflex could play an important modulatory role in maintaining neural and cardiovascular responses during low-intensity dynamic exercise [8, 26, 39]. Interestingly, MSNA decreases relative to that at rest during low-intensity leg cycling [18, 26, 43]. During dynamic exercise, intermittent compression of leg veins (i.e., muscle pump) elicits an increase in venous return and CBV, leading to the loading of the cardiopulmonary baroreceptors and consequently inhibits MSNA [8, 10, 26]. This sympathoinhibition during low-intensity exercise would buffer the ABP response and failure of such inhibition leads to an exaggerated ABP response [8]. To the best of our knowledge, it remains unclear what impact sex has on MSNA during low-intensity dynamic exercise, which could be related to the sympathoinhibitory effect of the cardiopulmonary baroreflex. In the present study, we did not find any sex differences in the sympathoinhibitory response induced by loading of the cardiopulmonary baroreceptors at rest. Therefore, it is plausible that the inhibition of MSNA during low-intensity dynamic exercise in young females also does not differ from that observed in age-matched males. Further studies are needed to validate this assumption.

## Conclusion

In the present study, we observed no differences in decreases in MSNA during the PLR test between young females and age-matched males. These results suggest that in young individuals, sex has minimal influence on the inhibition of sympathetic vasomotor outflow caused by the loading of the cardiopulmonary baroreceptors.

## Data Availability

The datasets used and/or analyzed during the current study are available from the corresponding author upon reasonable request.

## References

[1] Aneman A, Sondergaard S (2016). Understanding the passive leg raising test. Intensive Care Med.

[2] Badrov MB, Lalande S, Olver TD, Suskin N, Shoemaker JK (2016). Effects of aging and coronary artery disease on sympathetic neural recruitment strategies during end-inspiratory and end-expiratory apnea. Am J Physiol Heart Circ Physiol.

[3] Carter JR, Lawrence JE, Klein JC (2009). Menstrual cycle alters sympathetic neural responses to orthostatic stress in young, eumenorrheic women. Am J Physiol Endocrinol Metab.

[4] Cleroux J, Giannattasio C, Grassi G, Seravalle G, Sampieri L, Cuspidi C, Bolla G, Valsecchi M, Mazzola C, Mancia G (1988). Effects of ageing on the cardiopulmonary receptor reflex in normotensive humans. J Hypertens Suppl.

[5] Curry TB, Charkoudian N (2011). The use of real-time ultrasound in microneurography. Auton Neurosci.

[6] Davy KP, Seals DR, Tanaka H (1998). Augmented cardiopulmonary and integrative sympathetic baroreflexes but attenuated peripheral vasoconstriction with age. Hypertension.

[7] Delius W, Hagbarth KE, Hongell A, Wallin BG (1972). General characteristics of sympathetic activity in human muscle nerves. Acta Physiol Scand.

[8] Fadel PJ, Raven PB (2012). Human investigations into the arterial and cardiopulmonary baroreflexes during exercise. Exp Physiol.

[9] Fagius J, Wallin BG (1980). Sympathetic reflex latencies and conduction velocities in normal man. J Neurol Sci.

[10] Fisher JP, Young CN, Fadel PJ (2015). Autonomic adjustments to exercise in humans. Compr Physiol.

[11] Fu Q, Ogoh S (2019). Sex differences in baroreflex function in health and disease. J Physiol Sci.

[12] Fu Q, Okazaki K, Shibata S, Shook RP, VanGunday TB, Galbreath MM, Reelick MF, Levine BD (2009). Menstrual cycle effects on sympathetic neural responses to upright tilt. J Physiol.

[13] Fu Q, Sugiyama Y, Kamiya A, Shamsuzzaman AS, Mano T (1998). Responses of muscle sympathetic nerve activity to lower body positive pressure. Am J Physiol.

[14] Gagnon D, Schlader ZJ, Crandall CG (2015). Sympathetic activity during passive heat stress in healthy aged humans. J Physiol.

[15] Gauer OH, Sieker HO (1956). The continuous recording of central venous pressure changes from an arm vein. Circ Res.

[16] Grassi G, Giannattasio C, Cleroux J, Cuspidi C, Sampieri L, Bolla GB, Mancia G (1988). Cardiopulmonary reflex before and after regression of left ventricular hypertrophy in essential hypertension. Hypertension.

[17] Hinojosa-Laborde C, Chapa I, Lange D, Haywood JR (1999). Gender differences in sympathetic nervous system regulation. Clin Exp Pharmacol Physiol.

[18] Ichinose M, Saito M, Fujii N, Ogawa T, Hayashi K, Kondo N, Nishiyasu T (2008). Modulation of the control of muscle sympathetic nerve activity during incremental leg cycling. J Physiol.

[19] Joyner MJ, Wallin BG, Charkoudian N (2016). Sex differences and blood pressure regulation in humans. Exp Physiol.

[20] Katayama K, Barbosa TC, Kaur J, Young BE, Nandadeva D, Ogoh S, Fadel PJ (2020). Muscle pump-induced inhibition of sympathetic vasomotor outflow during low-intensity leg cycling is attenuated by muscle metaboreflex activation. J Appl Physiol.

[21] Katayama K, Dominelli PB, Foster GE, Kipp S, Leahy MG, Ishida K, Sheel AW (2021). Respiratory modulation of sympathetic vasomotor outflow during graded leg cycling. J Appl Physiol.

[22] Katayama K, Ishida K, Saito M, Koike T, Ogoh S (2016). Hypoxia attenuates cardiopulmonary reflex control of sympathetic nerve activity during mild dynamic leg exercise. Exp Physiol.

[23] Katayama K, Ishida K, Saito M, Koike T, Hirasawa A, Ogoh S (2014). Enhanced muscle pump during mild dynamic leg exercise inhibits sympathetic vasomotor outflow. Physiol Rep.

[24] Katayama K, Iwamoto E, Ishida K, Koike T, Saito M (2012). Inspiratory muscle fatigue increases sympathetic vasomotor outflow and blood pressure during submaximal exercise. Am J Physiol Regul Integr Comp Physiol.

[25] Katayama K, Kaur J, Young BE, Barbosa TC, Ogoh S, Fadel PJ (2018). High-intensity muscle metaboreflex activation attenuates cardiopulmonary baroreflex-mediated inhibition of muscle sympathetic nerve activity. J Appl Physiol.

[26] Katayama K, Saito M (2019). Muscle sympathetic nerve activity during exercise. J Physiol Sci.

[27] Katayama K, Saito M, Ishida K, Shimizu K, Shiozawa K, Mizuno S, Ogoh S (2022). Sympathetic vasomotor outflow during low-intensity leg cycling in healthy older males. Exp Physiol.

[28] Katayama K, Smith JR, Goto K, Shimizu K, Saito M, Ishida K, Koike T, Iwase S, Harms CA (2018). Elevated sympathetic vasomotor outflow in response to increased inspiratory muscle activity during exercise is less in young women compared with men. Exp Physiol.

[29] Lee JB, Notay K, Seed JD, Nardone M, Omazic LJ, Millar PJ (2021). Sex differences in muscle metaboreflex activation after static handgrip exercise. Med Sci Sports Exerc.

[30] Mancia G, Grassi G, Giannattasio C (1988). Cardiopulmonary receptor reflex in hypertension. Am J Hypertens.

[31] Mark AL, Mancia G (1983) Cardiopulmonary baroreflexes in humans. (ed) Handbook of Physiology, The Cardiovascular System, Peripheral Circulation and Organ Blood Flow. Am. Physiol. Soc., Bethesda, MD, p 795–813

[32] Mark AL, Victor RG, Nerhed C, Wallin BG (1985). Microneurographic studies of the mechanisms of sympathetic nerve responses to static exercise in humans. Circ Res.

[33] Millar PJ, Murai H, Morris BL, Floras JS (2013). Microneurographic evidence in healthy middle-aged humans for a sympathoexcitatory reflex activated by atrial pressure. Am J Physiol Heart Circ Physiol.

[34] Miller AJ, Cui J, Luck JC, Sinoway LI, Muller MD (2019). Age and sex differences in sympathetic and hemodynamic responses to hypoxia and cold pressor test. Physiol Rep.

[35] Minson CT, Halliwill JR, Young TM, Joyner MJ (2000). Influence of the menstrual cycle on sympathetic activity, baroreflex sensitivity, and vascular transduction in young women. Circulation.

[36] Morlin C, Wallin BG, Eriksson BM (1983). Muscle sympathetic activity and plasma noradrenaline in normotensive and hypertensive man. Acta Physiol Scand.

[37] Ng AV, Callister R, Johnson DG, Seals DR (1993). Age and gender influence muscle sympathetic nerve activity at rest in healthy humans. Hypertension.

[38] Notarius CF, Millar PJ, Doherty CJ, Incognito AV, Haruki N, O'Donnell E, Floras JS (2019). Microneurographic characterization of sympathetic responses during 1-leg exercise in young and middle-aged humans. Appl Physiol Nutr Metab.

[39] Ogoh S, Fisher JP, Fadel PJ, Raven PB (2007). Increases in central blood volume modulate carotid baroreflex resetting during dynamic exercise in humans. J Physiol.

[40] Rea RF, Hamdan M, Clary MP, Randels MJ, Dayton PJ (1985). Strauss RG (1991) Comparison of muscle sympathetic responses to hemorrhage and lower body negative pressure in humans. J Appl Physiol.

[41] Roddie IC, Shepherd JT, Whelan RF (1957). Reflex changes in vasoconstrictor tone in human skeletal muscle in response to stimulation of receptors in a low-pressure area of the intrathoracic vascular bed. J Physiol.

[42] Ryan KL, Rickards CA, Hinojosa-Laborde C, Cooke WH, Convertino VA (2012). Sympathetic responses to central hypovolemia: new insights from microneurographic recordings. Front Physiol.

[43] Saito M, Tsukanaka A, Yanagihara D, Mano T (1993). Muscle sympathetic nerve responses to graded leg cycling. J Appl Physiol.

[44] Tanaka H, Davy KP, Seals DR (1999). Cardiopulmonary baroreflex inhibition of sympathetic nerve activity is preserved with age in healthy humans. J Physiol.

[45] Taylor JA, Hand GA, Johnson DG, Seals DR (1992). Sympathoadrenal-circulatory regulation of arterial pressure during orthostatic stress in young and older men. Am J Physiol.

[46] Tripathi A, Mack GW, Nadel ER (1990). Cutaneous vascular reflexes during exercise in the heat. Med Sci Sports Exerc.

[47] Vallbo AB, Hagbarth KE, Torebjork HE, Wallin BG (1979). Somatosensory, proprioceptive, and sympathetic activity in human peripheral nerves. Physiol Rev.

[48] Victor RG, Leimbach WN (1987). Effects of lower body negative pressure on sympathetic discharge to leg muscles in humans. J Appl Physiol.

[49] Wallin BG, Thompson JM, Jennings GL, Esler MD (1996). Renal noradrenaline spillover correlates with muscle sympathetic activity in humans. J Physiol.

[50] Wenner MM, Greaney JL, Matthews EL, McGinty S, Kaur J, Vongpatanasin W, Fadel PJ (2022). Influence of age and estradiol on sympathetic nerve activity responses to exercise in women. Med Sci Sports Exerc.

[51] Yang H, Cooke WH, Reed KS, Carter JR (2012). Sex differences in hemodynamic and sympathetic neural firing patterns during orthostatic challenge in humans. J Appl Physiol.

